# Eco-Friendly Synthesis and Characterization of *Calotropis gigantea*-Derived Silver Nanoparticles for Combating Antibiotic-Resistant *Helicobacter pylori* and Gastric Cancer Cells

**DOI:** 10.3390/ph19030358

**Published:** 2026-02-25

**Authors:** Mounishwaran Kamalesan, Mohanraj Raja, Rameshkumar Neelamegam, Shashank S. Kamble, Douglas J. H. Shyu, Kayalvizhi Nagarajan

**Affiliations:** 1 Department of Zoology, School of Life Sciences, Periyar University, Salem 636011, Tamil Nadu, India; kmounish7@gmail.com (M.K.); rmohanrajzoology@gmail.com (M.R.); 2 Department of Biological Science and Technology, National Pingtung University of Science and Technology, Pingtung 912301, Taiwan; 3 Amity Institute of Biotechnology, Amity University, Mumbai 410206, Maharashtra, India; neelamegamramesh@gmail.com (R.N.); shashankcam@gmail.com (S.S.K.)

**Keywords:** *Helicobacter pylori*, silver nanoparticles (AgNPs), *Calotropis gigantea*, green synthesis, antibacterial activity, multifunctional therapeutic properties

## Abstract

**Background:** The eco-friendly synthesis of silver nanoparticles (AgNPs) utilizing medicinal flora presents a viable strategy for the development of multifunctional agents exhibiting antimicrobial, antioxidant, anti-inflammatory, and anticancer properties. This investigation aims to elucidate the phytochemical composition of *Calotropis gigantea* and its contribution to the synthesis of CG-AgNPs that demonstrate efficacy against *Helicobacter pylori* and gastric cancer cell lines. **Methods:** The aqueous plant leaf extract of *C. gigantea* underwent comprehensive analysis via gas chromatography-mass spectrometry (GC-MS), identifying a total of 25 bioactive constituents, including oleic and oxalic acid derivatives. The fabrication and analysis of silver nanoparticles (AgNPs) were performed utilizing methodologies including ultraviolet-visible (UV–Vis) spectroscopy, X-ray diffraction (XRD), Fourier-transform infrared spectroscopy (FTIR), scanning electron microscopy (SEM), energy-dispersive X-ray spectroscopy (EDX), high-resolution transmission electron microscopy (HR-TEM), dynamic light scattering (DLS), and assessments of zeta potential. Antibacterial efficacy was evaluated through methods including agar well diffusion, time-kill kinetics, and biofilm assays. The cytotoxic impact on AGS gastric cancer cells was investigated using MTT assays, DAPI staining, and acridine orange/ethidium bromide (AO/EtBr) staining techniques. The assessment of antioxidant potential was performed utilizing DPPH and ABTS assays. The anti-inflammatory properties were analyzed through protein denaturation and membrane stabilization tests. **Results**: CG-AgNPs exhibited a spherical morphology (11–17 nm) with commendable stability, denoted by using zeta potential analysis measurement of −30.2 mV. The antibacterial activity showed a significant inhibition zone of 16.00 ± 0.17 mm at a concentration of 50 µg/mL against *H. pylori*, in addition to notable biofilm disruption. The viability of AGS cells was reduced by 61% at a concentration of 100 micrograms per milliliter, with apoptosis being confirmed through relevant assays. The antioxidant potential varied from 18% to 83% (DPPH) and reached 74% (ABTS) at a concentration of 100 µg/mL. The anti-inflammatory assays indicated a BSA denaturation inhibition ranging from 45% to 80% and a membrane stabilization effect between 54% and 85%. **Conclusions:** CG-AgNPs exhibit substantial antibacterial, antioxidant, anti-inflammatory, and anticancer activities, underscoring their pharmaceutical potential, particularly for combating antibiotic-resistant pathogens and gastric malignancies.

## 1. Introduction

*Helicobacter pylori* represents a widely distributed pathogenic organism that infects approximately fifty percent of the global population, precipitating a range of gastrointestinal ailments, including peptic ulcer disease, primary gastritis, and gastric malignancies. This bacterium is characterized by its helical morphology, facultative anaerobic growth requirements, and Gram-negative classification. It can only survive in very difficult growth conditions [1]. The helical shape of *H. pylori* is thought to enable it to survive in the human gastrointestinal tract. Also, the helical shape of *H. pylori* facilitates bacterial movement, while the coccoid form allows colonization of gastric epithelial cells, thus promoting its invasiveness. Furthermore, *Helicobacter pylori* possesses the capability to establish biofilms, which contribute to the development of antimicrobial resistance, genetic alterations, and further complications in pathogen suppression [2].

*H. pylori* infection is predominantly contracted during childhood and, if not effectively eradicated, tends to persist as a chronic condition, frequently throughout one’s lifetime [3]. The successful eradication of *H. pylori*, evidenced by negative results from repeated diagnostic tests conducted at least four weeks post-therapy, is regarded as curative; however, the rates of successful eradication have been progressively declining over the years, primarily attributable to the development of bacterial resistance [4]. Amoxicillin, metronidazole, clarithromycin, tetracycline, and levofloxacin represent the most commonly utilized antibiotics, which are employed in various combinations within eradication protocols. The resistance exhibited by *H. pylori* to antibiotics is predominantly a result of mutations that are encoded chromosomally. The rise in multidrug-resistant *H. pylori* strains necessitates the formulation of alternative therapeutic approaches that are both efficacious and sustainable [5].

In recent times, the advancement of innovative, efficient nanotechnology-based antimicrobial agents targeting multidrug-resistant bacteria has emerged as a principal focus in biomedical research [6]. Nanomedicine has evolved into an interdisciplinary field that integrates principles from physics, chemistry, biology, and medicine to address human health issues. At present, the progress in the development of nanoparticle-based products is accelerating, with numerous formulations already available in the marketplace. Noble metal nanoparticles exhibit distinctive and markedly different physical and chemical characteristics compared to their macroscopic equivalents. As nanoparticles diminish in size, their surface-to-volume ratio, dispersion, and antimicrobial efficacy are significantly enhanced [7].

Various categories of metallic nanoparticles, including *Helianthus annuus* (sunflower) Au [8,9], *Calotropis gigantea* Ag [10], Oak Fruit Hull (Jaft) ZnO [11], and Oak Fruit Hull (Jaft) Cu [11], have been synthesized through biological methods utilizing bioagents such as plants, which typically demonstrate diminished toxicity towards eukaryotic cells. This distinctive feature offers promising prospects in the domain of biomedicine. The antimicrobial efficacy of metallic silver has been acknowledged for centuries, and it has been employed in a variety of medical apparatuses [12]. AgNPs are recognized for their capacity to induce both structural and physiological impairments in microbial cell membranes, resulting in modified membrane permeability and inhibition of respiratory proteins. These alterations compromise cellular homeostasis, ultimately culminating in the obliteration of microbial cells [13].

*Calotropis gigantea*, a flowering species within the Asclepiadaceae family, is indigenous to regions including Saudi Arabia, the northern region of Africa, the nation of Pakistan, the tropical regions of Africa, the western part of Asia, the state of Israel, and the Indo-Chinese territory. The foliar components of this species are replete with numerous bioactive compounds, encompassing toxic glycosides such as calotropin, uscharin, and calotoxin. Furthermore, the entirety of the plant has been conventionally utilized as both an anticoagulant and an anticancer therapeutic agent [14]. It has also been applied in the management of conditions such as asthma, otalgia, abdominal discomfort, arthritis, and dermatological disorders. Furthermore, the bioactive constituents found in the extract of *C. gigantea* have been further employed in the treatment of ailments, including somatic issues, sinusitis, diarrhea, fistulas, dermatological diseases, and jaundice [15].

In light of the escalating apprehension regarding antibiotic resistance in *Helicobacter pylori*, it is imperative to explore alternative therapeutic modalities that are not only efficacious but also sustainable. Nanotechnology, particularly the use of silver nanoparticles (AgNPs), has emerged as a potentially beneficial approach due to its potent antimicrobial attributes and capacity to overcome traditional resistance mechanisms. The green synthesis of AgNPs using *C. gigantea* presents an eco-friendly and cost-effective method that harnesses the plant’s bioactive compounds for enhanced antibacterial activity. This study aims to explore the synthesis, characterization, and multifunctional applications of *C. gigantea*-derived AgNPs, focusing on their potential to combat multidrug-resistant *H. pylori* and to offer a novel and sustainable solution in biomedical research.

## 2. Results

### 2.1. GC-MS Results

**Figure 1:**
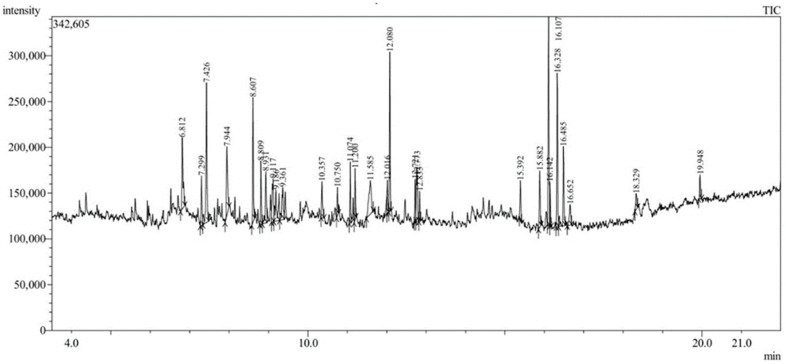
GC-MS chromatograph of *C. gigantea* plant leaf extracts.

The phytochemical components present in the aqueous extract derived from the leaf of *C. gigantea*, as identified by GC-MS analysis and presented in Table 1, revealed twenty-five compounds. GC–MS analysis of the aqueous leaf extract of *C. gigantea* revealed the presence of several bioactive constituents, including coumarin derivatives, long-chain hydrocarbons, esters, sugars, and triterpenoids such as α-amyrin, which are known to play a crucial role in nanoparticle reduction, stabilization, and biological activity (Figure 1).

### 2.2. Characterization of CG-AgNPs

**Figure 2:**
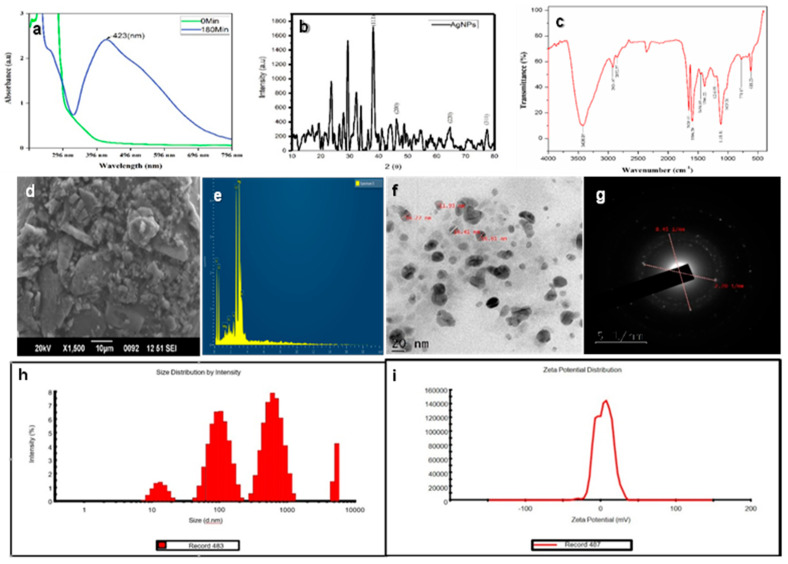
(**a**) UV–Visible spectrum of AgNPs synthesized from *Calotropis gigantea* leaf extract; (**b**) XRD; (**c**) FTIR; (**d**) SEM; (**e**) EDAX; (**f**) microscopy (HR-TEM); (**g**) SAED (**h**) DLS, (**i**) zeta potential.

The botanical specimen *C. gigantea* appears to represent a promising precursor of hydrocarbons. This flora is characterized by the presence of diverse organic constituents, including alkaloids, cardiac glycosides, tannins, flavonoids, sterols, and triterpenes. It is hypothesized that these constituents facilitate the reduction in AgNO_3_ to Ag [16]. The synthesized AgNPs were distinctly evidenced by the indication of a yellowish-brown shade, attributable to the excitation of surface plasmon resonance (SPR) for bands exhibiting a peak centered at approximately 423 nm (Figure 2a). The observed SPR phenomenon can be attributed to the abundance of free electrons in AgNPs, which also significantly influences the dimensions of the nanoparticles produced [17]. Numerous studies have posited that the surface plasmon resonance of AgNPs within the spectrum of 410–450 nm may be illustrative of the presence of spherical nanoparticles [18]. An XRD analysis was performed to elucidate the crystalline architecture and lattice attributes of the synthesized silver nanoparticles (AgNPs). The XRD pattern presented in Figure 2b reveals four prominent diffraction peaks at 2θ angles of 38.7°, 44.7°, 65.0°, and 77.8°. The thesis peaks correspond to the (111), (200), (220), and (311) crystallographic planes, respectively, thereby substantiating the face-centered cubic (FCC) configuration of the synthesized AgNPs. Furthermore, additional diffraction peaks were discerned within the XRD pattern, which are potentially attributable to biological constituents derived from the substance utilized in the synthetic methodology [19]. The FTIR spectrum of AgNPs is shown in (Figure 2c). FT-IR spectroscopy was conducted over the wavenumber range of 4000–400 cm^−1^, revealing a distinct absorption spectrum at 3426.01, 2924.07, 2852.37, 1596.39, 1456.65, 1216.09, 1025.20, 777.47, and 618.23 cm^−1^. The peaks at 3426.01 cm^−1^ correspond to O–H stretching vibrations, typically associated with hydrogen-bonded phenolic groups or alcohols. Peaks observed at 2924.07 and 2852.37 cm^−1^ are attributed to C–H stretching vibrations of alkanes, while the band at 1456.65 cm^−1^ is linked to CH_2_ and CH_3_ bending vibrations in alkanes. The absorption band at 1596.39 cm^−1^ is indicative of N–H bending of primary amines, and the peak at 1216.09 cm^−1^ corresponds to C–N stretching vibrations, further confirming the presence of amine groups. The band at 1025.20 cm^−1^ is assigned to the C–O stretching of carboxylic acids. Additionally, the peaks at 777.47 cm^−1^ and 618.23 cm^−1^ suggest the occurrence of C–Cl or C–Br stretching vibrations, characteristic of alkyl halides (Table 2).

These functional groups collectively suggest the involvement of biomolecules such as phenols, alcohols, amines, and carboxylic acids in the reduction and stabilization of AgNPs. The bioactive compounds in *C. gigantea*, such as flavonoids, phenols, ascorbic acid, and moderate levels of lycopene and β-carotene, contribute to its ability to reduce and cap nanoparticles. The phenols, in particular, play a crucial role in forming a strong coating on the nanoparticles, enhancing the capping process [20]. The structure of the biosynthesized AgNPs was examined utilizing scanning electron microscopy (SEM), which also provided insights into their particle size. The SEM images, shown in Figure 2d, reveal the size and structural characteristics of the synthesized AgNPs. The nanoparticles exhibit spherical, triangular, and cubic shapes. EDX analysis confirmed the presence of pure silver (Ag) in the synthesized nanoparticles, with a strong signal observed at 2.1 keV, accounting for 89.69% of the composition (Figure 2e). Additional elements detected included carbon (4.01%) and oxygen (6.31%). The notable presence of carbon and oxygen on the nanoparticle surface may be attributed to precursor impurities in the *C. gigantea* plant extract [21]. HR-TEM analysis further confirmed that the AgNPs demonstrated a consistent spheroidal morphology (Figure 2f). The particle size distribution ranged from approximately 11 to 17 nm, with an average size of 14.79 nm. It was elucidated that the nanoparticles did not exhibit direct contact, even when present in aggregates, thereby indicating efficient stabilization facilitated by a capping agent. This indicates the presence of biomolecules from the synthesis process, which help prevent agglomeration and enhance nanoparticle stability [22]. The selected-area electron diffraction (SAED) pattern (Figure 2g) displayed well-defined and pronounced concentric diffraction rings, thereby signifying the polycrystalline character of the synthesized AgNPs. The ascertained reciprocal lattice parameters of 8.45 nm^−1^ and 7.20 nm^−1^ are associated with interplanar spacings of approximately 0.118 nm and 0.139 nm, which are in substantial concordance with the distinct (220) and (200) crystallographic planes of face-centered cubic (FCC) silver, respectively. These observations are consistent with established crystallographic data (JCGDS card No. 04-0783), thereby validating the crystalline characteristics of the nanoparticles. Such SAED patterns are frequently documented in green-synthesized AgNPs, underscoring their elevated degree of atomic order and crystalline fidelity [23]. Moreover, dynamic light scattering (DLS) analysis uncovered a polydisperse size distribution, with intensity peaks spanning from tens to thousands of nanometers, thereby indicating the coexistence of both discrete nanoparticles and larger aggregates (Figure 2h). This extensive distribution is emblematic of the inherent variability in size arising from the intricate interactions of biomolecules present in the leaf extract, which acts as both a reducing and stabilizing agent. Polydispersity is characteristic of green synthesis approaches due to the heterogeneous nature of phytochemicals implicated in nanoparticle formation [24]. The zeta potential assessment revealed a peak value of approximately −30.2 mV, thereby suggesting that the AgNPs exhibit a significant negative surface charge (Figure 2i). This observation indicates remarkable colloidal stability, as the electrostatic repulsion among particles impedes aggregation. The negative surface potential can be attributed to the adsorption of phytoconstituents such as flavonoids, terpenoids, and phenolic acids derived from *C. gigantea* onto the nanoparticle surface, thereby enhancing both stabilization and biological functionality [25].

### 2.3. Antimicrobial Activity of CG-AgNPs Against H. pylori

**Figure 3:**
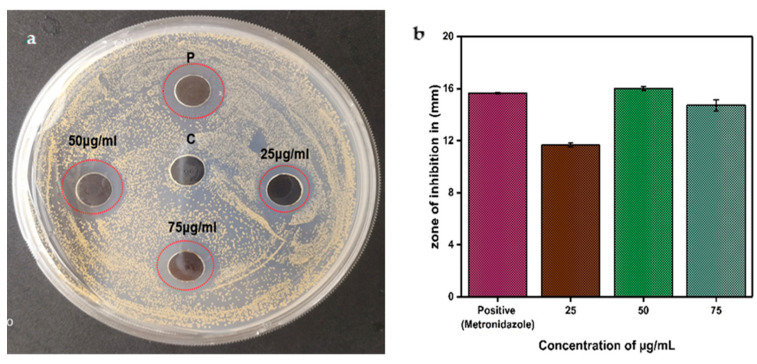
(**a**) Antimicrobial activity of CG-AgNPs against *H. pylori*; (**b**) a bar graph with error bars illustrating the analysis of antimicrobial activity data. P-Postive control (metronidazole (15 µg/mL) C-Control (DMSO).

Anti-*H. pylori* efficacy of silver nanoparticles (AgNPs) was quantitatively evaluated, demonstrating distinct zones of inhibition surrounding the wells containing various concentrations (25, 50, and 75 µg/mL) of nanoparticles, thus providing compelling evidence for the antibacterial characteristics of AgNPs. The inhibition zone identified at a concentration of 75 µg/mL was measured at 14.66 ± 0.52 mm, whereas the zones at 50 µg/mL and 25 µg/mL were recorded at 16.00 ± 0.17 mm and 11.66 ± 0.15 mm, respectively (Figure 3). At elevated concentrations (75 µg/mL), AgNPs tend to aggregate, which diminishes diffusion and results in a reduced inhibition zone. Conversely, at lower concentrations (50 µg/mL), AgNPs remain well-dispersed, facilitating improved diffusion and yielding a more substantial inhibition zone. Given that agar diffusion assays reflect diffusion efficacy rather than true antibacterial potency, the size of the inhibition zone may not necessarily increase in a strictly dose-dependent manner. A credible hypothesis regarding the mechanism of antibacterial efficacy is the liberation of Ag+ ions from silver nanoparticles (AgNPs), which can interfere with cellular proliferation through the establishment of robust interactions with thiol groups located in surface proteins, ultimately resulting in the lysis of bacterial cells [26]. In addition, AgNPs possess the capability to induce oxidative stress via the generation of reactive oxygen species (ROS), which can detrimentally impact enzymes and proteins, lea ding to irreversible harm to the DNA replication process [27].

### 2.4. Time Kill Assay

**Figure 4:**
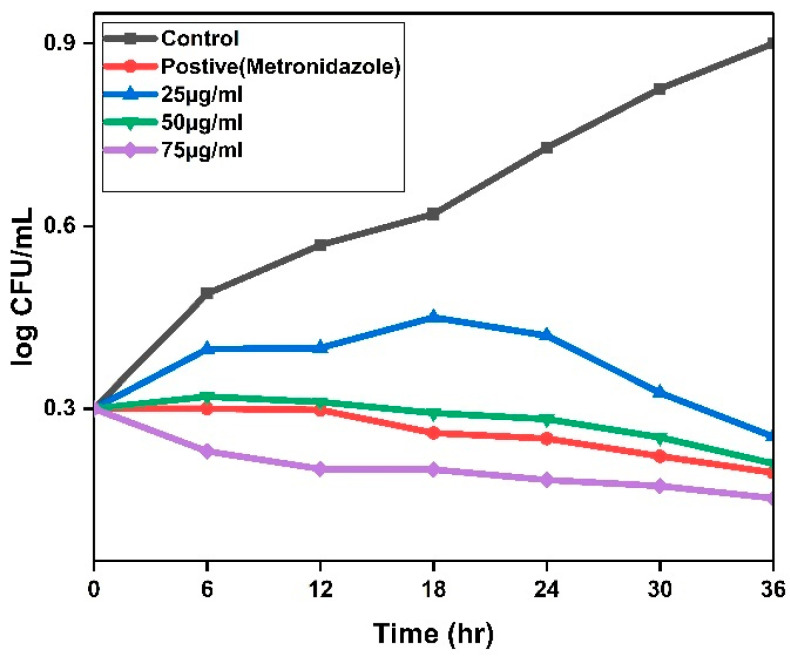
Time-kill assay of CG-AgNPs on *H. pylori* with 25 µg/mL, 50 µg/mL, and 75 µg/mL, for 36 h with regular 6 h gap intervals.

The time-kill kinetics assay showed that biosynthesized silver nanoparticles (AgNPs) demonstrated a bactericidal effect against *H. pylori* that is both concentration- and time-dependent. At the peak concentration evaluated (75 µg/mL), complete eradication of viable colonies (CFUs/mL) was accomplished within a 12 h exposure period. Conversely, the lowest concentration (25 µg/mL) necessitated 36 h to achieve 100% inhibition, whereas 50 ndµg/mL resulted in a significant reduction in bacterial viability after 24 h (Figure 4). Importantly, minimal reduction in CFUs was recorded at 25 µg/mL during the initial 24 h, implying that sub-lethal concentrations may prolong bacterial clearance. These findings indicate that elevated concentrations of AgNPs expedite bactericidal activity, aligning with previous investigations that demonstrated AgNPs disrupt bacterial membrane integrity, stimulate oxidative stress induced through the formation of ROS, and interfere with crucial cellular functions [28,29]. The pronounced decline in bacterial viability, concomitant with increasing nanoparticle concentration, underscores the formidable antimicrobial properties of AgNPs, likely attributable to their nanoscale dimensions and extensive surface area that promote direct interactions with bacterial cells [30]. Comparable time-dependent effects have also been documented for AgNPs against other Gram-negative bacteria, including *Escherichia coli* and *Pseudomonas aeruginosa*, thereby reinforcing their broad-spectrum antimicrobial potential [31]. Collectively, the data advocate for the utilization of plant-derived AgNPs as a promising antimicrobial approach against *H. pylori*, particularly in light of the escalating challenge posed by antibiotic resistance. The optimization of dosage and exposure duration could further enhance their therapeutic efficacy.

### 2.5. Determination of Biofilm Activity

**Figure 5:**
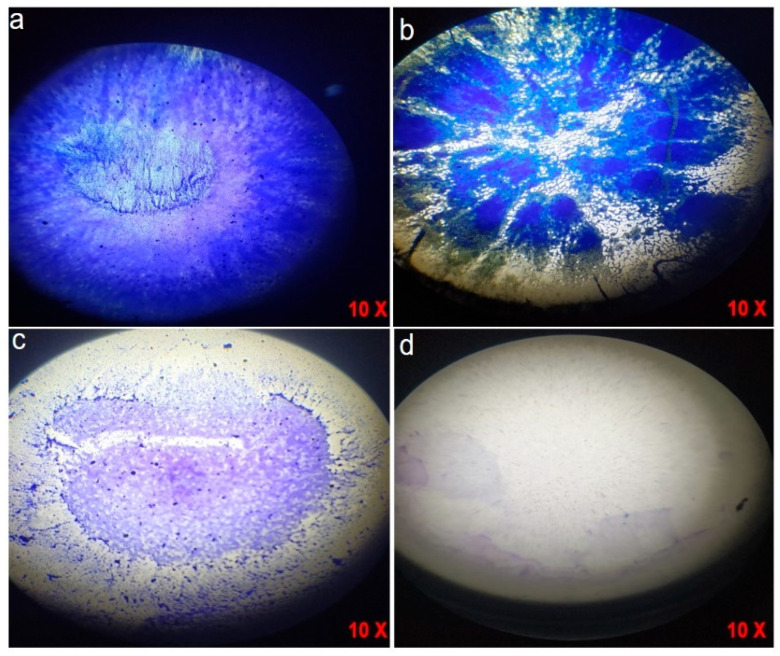
Determination of biofilm activity using crystal violet of CG-AgNPs against *H. pylori* anti- biofilm activity. (**a**) Control; (**b**) 25 µg/mL; (**c**) 50 µg/mL; (**d**) 75 µg/mL. The microscopic magnification power of the images 10X.

Crystal violet staining elucidated a concentration-dependent suppression of *H. pylori* biofilm formation following administration of CG-AgNPs. In the untreated control (Figure 5a), a dense and well-organized biofilm matrix was observed, thereby indicating the formidable biofilm-forming capacity of *H. pylori*. Treated with 25 µg/mL of CG-AgNPs exhibited partial disruption, characterized by apparent zones of thinning and diminished biomass (Figure 5b). A more significant inhibition of biofilm formation was noted at 50 µg/mL (Figure 5c), resulting in a considerable reduction in biofilm density and coverage. Remarkably, at the highest concentration of 75 µg/mL (Figure 5d), minimal to no detectable staining was observed, signifying near-complete inhibition of biofilm development. These findings imply that CG-AgNPs exert a potent antibiofilm effect against *H. pylori*, likely through interference with the bacterial adhesion mechanism, extracellular polymeric substance (EPS) production, and quorum-sensing pathways [32]. The antibiofilm efficacy may also be ascribed to the diminutive size and elevated surface reactivity of the AgNPs, which facilitate their penetration into the biofilm matrix and disrupt microbial consortia [27]. Prior investigations have similarly indicated that green-synthesized AgNPs possess the capability to inhibit biofilms of various pathogens by inducing oxidative stress, compromising bacterial membrane integrity, and downregulating biofilm-associated genes [29,33]. In light of the escalating antibiotic resistance observed in *H. pylori*, the capacity of CG-AgNPs to disrupt biofilms presents a promising avenue for adjunctive or alternative anti-*H. pylori* therapeutic strategies. This biofilm inhibition augments the exposure of bacteria to host immune defenses and therapeutic agents, thereby enhancing eradication success rates.

### 2.6. Antibiofilm Activity of H. pylori of SEM Image

**Figure 6:**
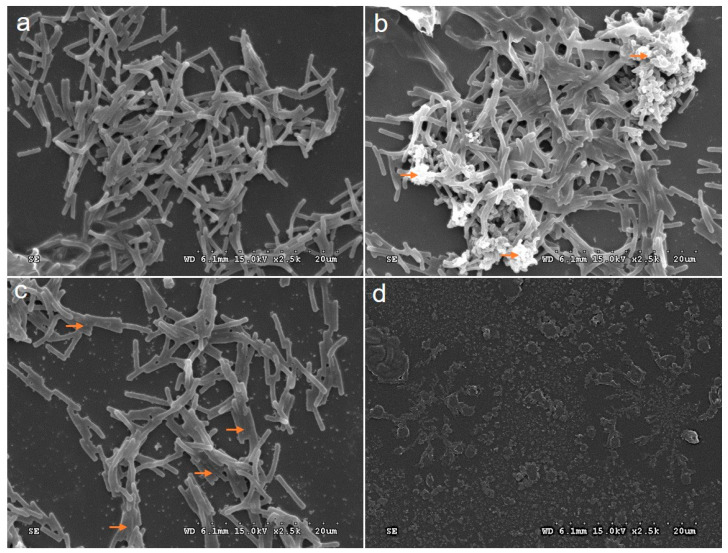
SEM image analysis of CG-AgNPs against *H. pylori* antibiofilm activity. (**a**) Control; (**b**) 25 µg/mL; (**c**) 50 µg/mL; (**d**) 75 µg/mL. The orange arrows indicate cells showing a stressed membrane.

The exploration of the antibiofilm efficacy of CG-AgNPs against *H. pylori* represents a burgeoning field of inquiry. The objective of this experimental study was to elucidate the capacity of CG-AgNPs to impede biofilm formation by *H. pylori*. In a microtiter plate, the analytical approach was applied to evaluate the influence of CG-AgNPs on the inhibition of biofilm development. *H. pylori* strains were cultivated for a duration of 24 h within the microtiter plate wells and subsequently subjected to treatment with CG-AgNPs at a concentration of 25 to 75 μg/mL (refer to Figure 6). The administration of *H. pylori* with 75 μg/mL of AgNPs for a period of 24 h resulted in a notable reduction in biofilm activity. SEM images of the control showed uniform biofilm architecture with dense bacterial accumulations and long fibrils connecting cells. The bacterial morphology changed from bacillary to coccoid in older cultures, with fiber-like structures adhering to rod-shaped bacteria in the biofilms. However, in the treated biofilm, the formation of *H. pylori* was decreased with increased concentration. An environmentally friendly method for synthesizing CG-AgNPs and explored their dose-dependent ability to inhibit biofilm formation by the human pathogen *H. pylori* [34]. It has been established that, within these bacterial populations, the activity of biofilms exhibits greater sensitivity to silver nanoparticles (AgNPs) than the phenomenon of cellular mortality, indicating that separate signaling pathways may be implicated in the processes of cellular survival and biofilm development.

### 2.7. Anti-Cancer Assay

#### 2.7.1. MTT Assay

**Figure 7:**
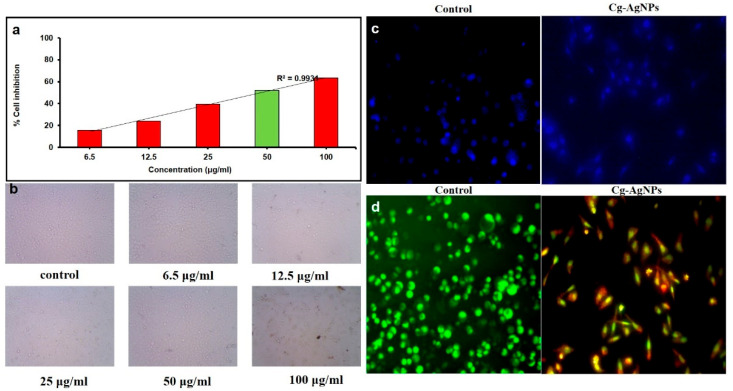
Anti-cancer activities. (**a**,**b**) MTT assay; (**c**) DAPI staining; (**d**) AO/EtBr staining against CG-AgNPs. The cells were examined using a fluorescent microscope in (40X).

To assess the cytotoxic effects of AgNPs on eukaryotic cells, MTT assays were performed to evaluate their biocompatibility with AGS gastric epithelial cells, a widely recognized model for *H. pylori* infection [35]. At the highest concentration assessed during the evaluation of 100 μg/mL cell inhibition was 61%. At concentrations of 100 μg/mL, 50 μg/mL, 25 μg/mL, 12.5 μg/mL, and 6.5 μg/mL, the inhibition was 61%, 58%, 41%, 37%, and 26%, respectively (Figure 7a,b), compared to control cells. The evaluation of cell inhibition with varying concentrations of CG-AgNPs demonstrated a concentration-dependent effect on AGS cell viability. Earlier studies reported that cancer cell viability decreased with increasing nanoparticle concentration, which closely aligns with the results of the current study [36]. Similarly, the synthesized py-AgNPs also possess potent anti-cancer effects against AGS cells, highlighting their promise as candidates for novel cancer drug development [37]. The toxicity of AgNPs was impacted by aspects like the size of the particles, shape, concentration, and synthesis method, with smaller nanoparticles (5 nm) exhibiting greater toxicity compared to larger ones (20–50 nm) [38].

#### 2.7.2. DAPI Staining

DAPI staining of AGS gastric carcinoma cells treated with CG-AgNPs revealed marked nuclear morphological changes compared to untreated controls. In the control group, cells displayed intact, rounded, and evenly stained nuclei, indicating healthy, viable cells with no signs of nuclear condensation or fragmentation (Figure 7c). In contrast, the CG-AgNP-treated group exhibited nuclear condensation and a reduction in the number of intact nuclei, suggestive of early apoptotic events. The DAPI signal appeared more diffused and irregular in intensity, reflecting possible chromatin condensation or DNA fragmentation, both hallmarks of apoptosis [39]. These findings are supported by previous studies reporting that AgNPs induce apoptosis in cancer cells via oxidative stress, mitochondrial dysfunction, and activation of caspase-mediated pathways [40,41]. The observed nuclear changes indicate that CG-AgNPs may exert their cytotoxic effects by triggering programmed cell death in AGS cells. Furthermore, this observation aligns with the hypothesis that plant-derived AgNPs serve as effective anti-cancer agents due to their enhanced biocompatibility and reduced systemic toxicity [41]. Complementary crystal violet staining showed reduced cell density and disrupted monolayer integrity in the treated group, further confirming cytotoxicity. This indicates a dual action of CG-AgNPs—direct nuclear damage and compromised membrane integrity—leading to effective anti-proliferative activity in gastric cancer cells. Together, the DAPI and crystal violet results provide strong evidence that CG-AgNPs can inhibit AGS cell viability through induction of apoptotic pathways.

#### 2.7.3. AO/EtBr Staining

Dual staining utilizing acridine orange (AO) and ethidium bromide (EtBr) afforded visual confirmation of apoptosis induction in AGS cells exposed to CG-AgNPs. In the control cohort, cells manifested uniform green fluorescence, signifying intact membranes and viable nuclei, which are characteristic of healthy, non-apoptotic cellular states. Conversely, the group treated with CG-AgNPs exhibited a spectrum of yellow-orange to red fluorescence, with numerous cells presenting condensed or fragmented nuclei, indicative of early and late stages of apoptosis, respectively. AO permeates both viable and non- viable cells, emitting green fluorescence upon intercalation with double-stranded DNA, whereas EtBr selectively infiltrates cells with compromised membranes, imparting an orange to red hue to the nucleus contingent upon the phase of cellular demise (Figure 7d) [42]. The observed chromatic transition in the treated cells, from green to orange-red, signifies the gradual apoptotic transformations instigated by the nanoparticles. The morphological modifications and fluorescence characteristics robustly suggest that CG-AgNPs precipitate membrane destabilization and nuclear impairment, potentially instigating caspase-mediated apoptosis [38]. Similar to previous investigations that elucidate the capacity of AgNPs to induce apoptosis via the generation of reactive oxygen species (ROS), loss of mitochondrial membrane potential, and cell cycle arrest [42,43]. The pronounced apoptotic response documented herein further substantiates the therapeutic potential of green-synthesized AgNPs in targeting gastric cancer cells, accentuating their applicability as alternative or supplementary anticancer agents.

### 2.8. Antioxidant Assay

The antioxidant activity of synthesized silver nanoparticles (CG-AgNPs) was evaluated using both DPPH and ABTS free radical scavenging assays.

#### 2.8.1. DPPH Assay

**Figure 8:**
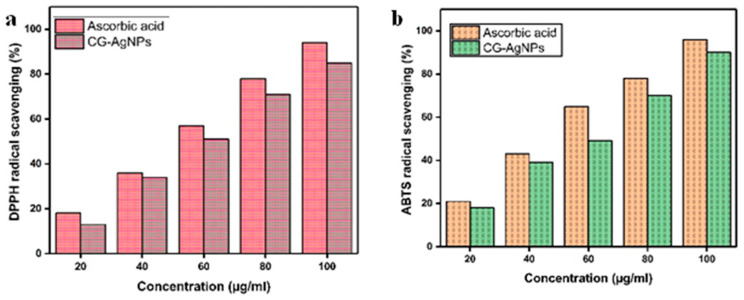
Antioxidant activities. (**a**) DPPH assay and (**b**) ABTS scavenging assay against Cg-AgNPs.

DPPH is a stable free radical commonly used to assess antioxidant capacity due to its ability to accept hydrogen atoms or electrons. The CG-AgNPs exhibited significantly higher DPPH scavenging activity in a dose-dependent manner, with scavenging percentages ranging from 18% to 83% at concentrations of 20 to 100 µg/mL. Despite this, the antioxidant activity of CG-AgNPs remained lower than that of the standard ascorbic acid, which showed 90% scavenging at 100 µg/mL (Figure 8a). Similarly Sriram et al. [44] synthesized AgNPs exhibited dose-dependent antioxidant activity in DPPH assays, with higher scavenging efficiency (IC_50_ = 0.70 ± 0.08 mg/mL) than the extract but lower than ascorbic acid (74% at 1 mg/mL). However, *Persea americana* fruit peel extract-mediated silver nanoparticles (AgNPs) exhibited DPPH free radical scavenging activities ranging from 57.82% to 63.25%, which may be attributed to the presence of biomolecules adhered to the surface of the CG-AgNPs [45].

#### 2.8.2. ABTS Scavenging Assay

ABTS represents a stable radical cation that possesses the ability to donate protons. At a concentration of 100 µg/mL, the percentage inhibition values for CG-AgNPs and the standard were recorded at 74% and 80%, respectively. All experimental samples demonstrated a dose-dependent response in antioxidant activity (Figure 8b). These results indicate that the standard demonstrated stronger antioxidant activity compared to the AgNPs. Previous reports stated dose-dependent ABTS radical scavenging activity, with AgNPs synthesized using leaf extract (IC_50_: 304.31 μg/mL) showing greater efficacy than those synthesized using stem extract (IC_50_: 326.83 μg/mL) [46]. Das et al. [47] reported that green-synthesised AgNPs produced using *Ipomoea batatas* (L.) Lam peel extract shows the ABTS scavenging activity of Ib1-AgNPs with the IC_50_ of 327.41 μg/mL). The antioxidant potential of AgNPs may be attributed to the diverse phytochemicals in the plant extract, particularly alkaloids, tannins, and saponins, which exhibit significant free radical scavenging and reducing power by effectively quenching ROS through their oxidizing and reducing capabilities [48].

### 2.9. Anti-Inflammatory Activity

**Figure 9:**
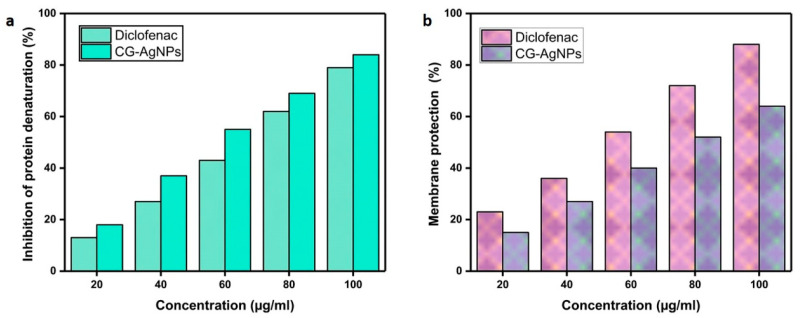
Anti-inflammatory activities. (**a**) Inhibition of protein denaturation and (**b**) membrane stability assay against CG-AgNPs.

Inflammation serves as a key immune response to foreign particles, involving the activation of immune cells and the release of pro-inflammatory cytokines. Although CG-AgNPs have shown potential in mitigating inflammation, their anti-inflammatory efficacy remains relatively limited [49]. *C. gigantea* mediated AgNPs demonstrated significant inhibitory effects on BSA denaturation, with a dose-dependent response similar to a standard anti-inflammatory drug (Figure 9a). At 20 µg/mL, the AgNPs achieved 45% inhibition, gradually increasing to 80% at 100 µg/mL, while the standard drug showed comparable inhibition (47% at 10 µg/mL, 84% at 50 µg/mL). These results suggest that the AgNPs possess anti-inflammatory potential, making them a promising natural alternative to synthetic anti-inflammatory drugs. In the membrane stabilization assay (Figure 9b), AgNPs mediated by *C. gigantea* exhibited disparate degrees of membrane stabilization at varying concentrations, demonstrating effects that are analogous to the standard anti-inflammatory pharmaceutical agent, diclofenac sodium. When at a volume of 20 µg/mL, the AgNPs achieved a stabilization percentage of 54%, whereas diclofenac sodium recorded a stabilization rate of 58%. The degree of stabilization was observed to escalate with increasing concentration, culminating in 85% for AgNPs and 89% for diclofenac sodium at a concentration of 100 µg/mL. These findings imply that the AgNPs possess anti-inflammatory properties through their capacity to stabilize cellular membranes, exhibiting effects that are comparable to those of diclofenac sodium across all concentrations evaluated. In a similar vein, Prabakaran et al. [50] reported that AgNPs manifested considerable in vitro anti-inflammatory activity against protein denaturation, achieving an inhibition rate of 88.16% at a concentration of 500 µg/mL. This activity was found to be comparable to that of diclofenac sodium, which demonstrated an inhibition rate of 89.65% at the identical concentration. The effective anti-inflammatory activity of AgNPs may be attributed to their enhanced permeability and absorption characteristics, which could help inhibit edema formation in cells, potentially through the binding of anti-inflammatory bioactive compounds on their surface [51,52].

## 3. Materials and Methods

### 3.1. Study Site and Sampling of Plant Leaf Collection

**Figure 10:**
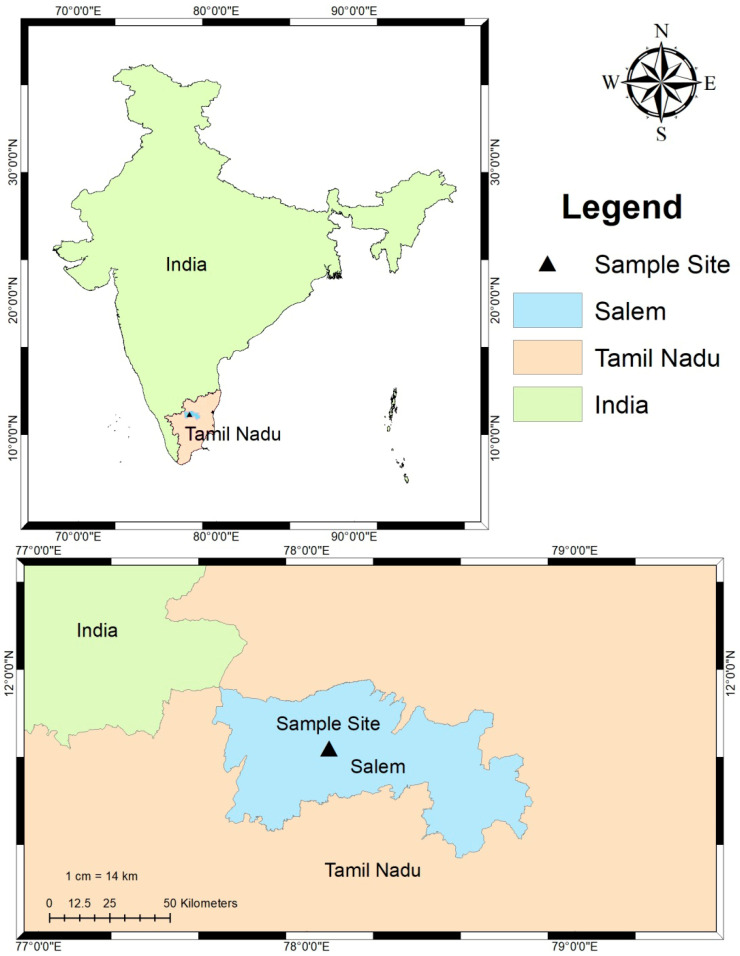
Study site and sampling of plant leaf collection of *C. gigantea*.

In January 2022, healthy foliar specimens of *C. gigantea* (family: Apocynaceae) were meticulously gathered from Salem, Tamil Nadu, India, to investigate its ecological resilience and potential biotechnological applications [53]. The designated sampling locations encompassed Site, Periyar University Botanical Garden (11°42′26.1′′ N, 78°04′13.9′′ E), characterized by red loamy soil. The harvested leaves were securely placed in sterile bags and preserved at a temperature of 4 °C until further analytical procedures could be conducted. A cartographic representation of the sampling sites and corresponding plant photographs is illustrated in Figure 10.

### 3.2. Preparation of Plant Extract

Fresh leaves of *C. gigantea* (CGL) were meticulously harvested and subsequently subjected to thorough washing with DH_2_O in order to eliminate any dust and extraneous impurities, and the plant was authenticated (PU/BOT/2024/038) by the Department of Botany, Periyar University. Following this procedure, the leaves were shade-dried at ambient temperature (22 °C) for a duration of two weeks, thereby preserving the integrity of the bioactive compounds. The dried material was finely powdered using a mechanical grinder. An aggregate of 10 g of finely powdered desiccated foliage was subsequently placed into a 500 mL beaker with 100 mL of DH_2_O, and the resulting mixture was subjected to boiling at a temperature of 100 °C for a timeframe of 20 min. Following the cooling of the solution to ambient temperature, the contents of the beaker were filtered utilizing Whatman Grade 1 filter paper [54,55]. The resultant leaf extract was then concentrated under reduced pressure and stored at a temperature of 4 °C for subsequent analyses.

### 3.3. GC-MS Analysis

GC-MS analysis was conducted using a GC-MS-QP 2010 SE (Shimadzu, Tokyo, Japan) instrument. A fused silica column packed with elite-5MS (5% biphenyl 95% dimethylpolysiloxane, 30 × 0.25 mm ID 250 µm df) and helium as carrier gas at a constant flow rate of 1 mL/min was used to separate the components. The specification of constituents was established through a comparative review of their mass spectra relative to the archives of the Wiley and NIST libraries, as articulated by [56], coupled with an appraisal of their retention indices as indicated in the prevailing literature. The identification of compounds within the crude extract has been ascertained through the analysis of GC–MS retention time.

### 3.4. Synthesis of AgNPs Using C. gigantea

Biogenic AgNPs were synthesized utilizing the aqueous extract of *C. gigantea* as a reductant, capping agent, and stabilizing component. In summary, the 10 mL of the aqueous plant leaf extract was combined with 90 mL of a 1 mM silver nitrate solution to facilitate the biosynthesis of AgNPs, which were subsequently stirred in a dark environment for a duration of 3 h at ambient temperature to inhibit auto-oxidation. The transition in color from a colorless silver nitrate solution to a dark brown hue served as an indication of AgNP synthesis, further corroborated by UV spectrophotometric analysis. The synthesized silver nanoparticles (AgNPs) were subjected to centrifugation of 15 min at a rotational speed of 15,000 rpm utilizing a Remi centrifuge (CM-12 plus) to yield compact pellets of colloidal silver nanoparticles (CG-AgNPs), which were subsequently redispersed in demineralized water to eliminate any residual contaminants and unreduced silver nitrate, followed by a drying process and storage at ambient temperature for subsequent characterization.

### 3.5. Characterization of CG-AgNPs

The synthesis of AgNPs was corroborated through the application of Ultraviolet-visible spectral analysis. The absorbance spectra were diligently acquired utilizing the U-vis spectroscopy (UV-1800 Shimadzu UV spectrophotometer ver 2.50, Shimadzu Corporation, Kyoto, Japan) across a wavelength range extending from 200 to 800 nm. FTIR was conducted utilizing a (PerkinElmer Spectrum 2000, PerkinElmer, Inc., Waltham, MA, USA) Spectrometer to determine the possible functional groups that are present in the biomolecules extracted from the plant source. The XRD analysis was performed utilizing an XRD (Shimadzu—6000, Shimadzu Corporation, Japan) operating at 30 kV and 100 mA, with spectra captured employing CuKα radiation at a wavelength of 1.5406 Å over the 2θ range of 20° to 80°. The surface morphology and dimensions of the AgNPs were investigated through SEM coupled with EDX analysis on the Hitachi 4500 instrument (Hitachi, Ltd., Tokyo, Japan), alongside TEM and SAED assessments on a Hitachi H-7500 instrument. The characterization of the size distribution of nanoparticles and the surface charge of AgNPs was conducted utilizing a particle size analyzer (Zetasizer nano ZS, Malvern Instruments Ltd., Worcestershire, UK) at a controlled temperature of 25 °C and employing a detection angle of 90°.

### 3.6. Antimicrobial Activity of CG-AgNPs Against H. pylori

The anti-*H. pylori* efficacy was assessed through the implementation of a well diffusion assay.

With minimal modifications, a culture of *H. pylori* (Gram-negative) was meticulously prepared and subsequently inoculated onto Brucella agar plates that had been enriched with 7% sheep blood, which were then allowed to solidify. The antibacterial efficacy of the synthesized CG-AgNPs against *H. pylori* was evaluated utilizing the agar well diffusion technique, as previously described with slight adjustments [57]. A bacterial suspension adhering to the 0.5 McFarland standard (~1 × 10^8^ CFU/mL) was carefully applied across the agar plate surface using a sterile cotton swab. Wells, each with a diameter of 6 mm, were formed in the agar using a sterile cork borer, into which 100 µL of CG-AgNP solution at varying concentrations (25, 50, and 75 µg/mL) was introduced into each well. A well containing DMSO served solely as the control, while metronidazole (15 µg/mL) was utilized as the positive control. The plates were incubated under microaerophilic conditions at 37 °C for a period of 72 h. The zones of inhibition were measured in millimeters.

### 3.7. Time Kill Assay

The bactericidal efficacy of CG-AgNPs was assessed through the analysis of killing kinetics. *H. pylori* was propagated in the aforementioned liquid medium and concurrently exposed to CG-AgNPs at a concentration corresponding to the minimum bactericidal concentration (MBC). Following incubation periods of 0, 6, 12, 18, 24, 30, and 36 h, the quantification of colony-forming units (CFUs) was conducted by performing serial dilutions of each sample and plating them onto designated agar plates. The rate of bacterial lethality was represented as a viable count (log_10_ CFU/mL) over time. A control experiment devoid of CG-AgNPs was also executed.

### 3.8. Determination of Biofilm Activity Using the Tissue Culture Plate Method

The inhibition of biofilm formation was assessed in accordance with previously established protocols, with appropriate modifications implemented. In summary, the bacterial cells were cultivated in Brucella broth enriched with 2% Fetal Bovine Serum (FBS), and individual wells of sterile 6-well flat-bottom polystyrene tissue culture plates (TCPs) were inoculated with 5 mL of a singular bacterial species at a concentration of 1 × 10^6^/mL. Subsequently, the cell culture plates were incubated with AgNPs for 24 h at 37 °C. Subsequent to the incubation period, the culture media were discarded, and the wells underwent three wash cycles utilizing 5 mL of sterile distilled water in order to remove non-adherent bacterial cells. A crystal violet solution, appropriately diluted in water, was then applied to each well for a period of 45 min. The wells were subsequently subjected to five rinses with 6 mL of sterile DH_2_O to eliminate any residual staining agent. The optical density of each well was measured at an absorbance wavelength of 595 nm utilizing a microtiter ELISA reader (Bio-Rad Laboratories, Inc., Hercules, CA, USA) [33,58].

### 3.9. Determination of Biofilm Activity Using SEM

The assessment of biofilm formation by *Helicobacter pylori* was executed through the cultivation of the bacterium in Brain Heart Infusion (BHI) broth enriched with 10% fetal bovine serum (FBS) under microaerophilic conditions, followed by exposure to AgNPs at concentrations of 25, 50, and 75 µg/mL, with untreated cultures utilized as controls. Sterile glass coverslips positioned within 24-well plates were employed for the promotion of biofilm development over a duration of 72 h. Subsequent to incubation, coverslips were meticulously washed with phosphate-buffered saline (PBS), fixed using 2.5% glutaraldhyde, dehydrated via a graded series of ethanol, dried, and subjected to gold sputter-coating. SEM imaging was conducted to examine the morphological characteristics of the biofilm and any structural alterations, while ImageJ (https://imagej.net/ij/, accessed on 22 February 2026; National Institutes of Health, Bethesda, MD, USA); was utilized for quantification of surface area coverage. This methodology was adapted from established protocols with minor modifications to accommodate nanoparticle treatments [59,60].

### 3.10. Anticancer Activity

#### 3.10.1. MTT Assay

The experimental investigation was undertaken to clarify the cytocompatibility of the synthesized silver nanoparticles (AgNPs). The AGS cell lines were procured from the National Center for Cell Sciences (NCCS, Pune). The cellular culture was sustained in a humidified environment with a CO_2_ concentration of 50 g/mL at a regulated temperature of 37 °C, employing Dulbecco’s modified Eagle’s medium (DMEM) supplemented with 10% fetal bovine serum (FBS), penicillin (100 U/mL), and streptomycin (100 g/mL). In 24-well plates, AGS cells (approximately 1 × 10^5^ cells per well) were inoculated and incubated at 37 °C within a CO_2_-enriched atmosphere. The synthesized AgNPs were prepared in varying concentrations of 6.5, 12.5, 25, 50, and 100 µg/mL and subsequently incubated for durations of 24, 48, and 72 h at 37 °C. Subsequently, the cells underwent treatment for a period of 4 h with 3-(4,5-dimethylthiazol-2-yl)-2,5-diphenyl tetrazolium bromide (MTT). To facilitate the solubilization of formazan crystals, 1 mL of DMSO was introduced, and optical density (OD) measurements were ascertained utilizing an ELISA reader Bio-Rad 680 (Bio-Rad Laboratories, Inc., USA) at 570 nm, and the experiment was carried out in triplicate, and the percentage of viable cells was calculated employing the following formula:


Cell Viability % = (*A*_570_ of treated cells)/(*A*_570_ of control Cells) × 100

The cellular specimens exposed to varying concentrations of silver nanoparticles (AgNPs) were preserved utilizing 4% paraformaldehyde and subsequently analyzed through optical microscopy [61].

#### 3.10.2. Apoptotic Effects Assessed via AO/EtBr and DAPI Staining

##### 3.10.2.1. AO/EtBr

Morphological alterations associated with apoptosis in AGS gastric carcinoma cells subjected to treatment with CG-AgNPs were evaluated utilizing acridine orange/ethidium bromide (AO/EtBr) dual fluorescent staining, in accordance with the methodology delineated by [62], albeit with minor modifications. In summary, AGS cells were inoculated at a density of 5 × 10^5^ cells/mL into 24-well tissue culture plates and allowed to incubate overnight at 37 °C under a 5% CO_2_ atmosphere to facilitate cellular adherence. Subsequently, the cells were administered a treatment of 42.55 μg/mL of CG-AgNPs suspended in serum-free DMEM and incubated for a duration of 24 h. Following the treatment, 50 µL of a fluorescent dye mixture composed of acridine orange and ethidium bromide (1 mg/mL each, prepared in phosphate-buffered saline) was introduced into each well. The contents of the wells were gently mixed and incubated in a dark environment for a period of 5 min. The plate was thereafter subjected to centrifugation at 800 rpm for 2 min to allow the stained cells to settle. The stained cells were promptly examined under a fluorescence microscope equipped with an appropriate filter set (excitation: 480–490 nm; emission: >515 nm). A minimum of 100 cells per treatment group were scrutinized to assess the occurrence of apoptosis. Viable cells exhibited green fluorescence with uniform chromatin; early apoptotic cells displayed green fluorescence accompanied by condensed or fragmented chromatin, and late apoptotic or necrotic cells were stained orange to red, indicating disrupted nuclei.

##### 3.10.2.2. DAPI (4′,6-Diamidino-2-Phenylindole) Staining for Nuclear Apoptosis

For the purpose of DAPI staining to assess nuclear apoptosis, the cells were similarly cultured on a glass coverslip in a 24-well plate and subsequently exposed to the compound for a duration of 24 h. The fixed cells underwent permeabilization utilizing 0.2 percent Triton X-100 (50 µL) for a period of 10 min at ambient temperature, followed by incubation for 3 min with 10 µL of DAPI, employing a coverslip to facilitate uniform distribution of the stain. The cellular samples were analyzed using a fluorescent microscope (Nikon Eclipse, Inc., Tokyo, Japan) with excitation and emission wavelengths set at 359 nm and 461 nm, respectively.

### 3.11. Evaluation of Antioxidant Assay

#### 3.11.1. DPPH Assay

The antioxidant properties of the synthesized CG-AgNPs were evaluated utilizing the DPPH radical scavenging assay. CG-AgNPs, prepared at various concentrations of 20, 40, 60, 80, and 100 μg/mL, were meticulously agitated and combined with a 0.1 mM solution of DPPH. The resultant mixture was subsequently incubated in the absence of light for a duration of 30 min. The reduction in the concentrations of DPPH was assessed by measuring the absorbance at a wavelength of 517 nm [63]. Subsequently, the antioxidant capacity of CG-AgNPs was quantified using ascorbic acid as the reference standard. The DPPH scavenging efficacy was expressed as a percentage, calculated in the following manner:


DPPH scavenging assay (%) = (Control Absorbance − (Sample Absorbance − Blank Absorbance))/(Control Absorbance) × 100

#### 3.11.2. ABTS Radical Scavenging Assay

The ABTS·+ radicals were synthesized via the interaction of potassium persulfate (2.5 mM) with ABTS (7 mM) at a 1:1 volume ratio and were thereafter preserved in a controlled dark environment at a constant temperature of 25 °C for a period ranging from 10 to 15 h preceding their application. The reagent solution underwent a dilution process with methyl alcohol until an absorbance of 0.700 was attained at a wavelength of 734 nm. Silver nanoparticles and/or extract (5 µL) were combined with ABTS solution (4 mL), and after an incubation period of 30 min, absorbance was assessed. All antioxidant assays were conducted in triplicate to ensure the robustness of the results. Ultimately, the percentage of radical-scavenging activity was computed [64].


ABTS scavenging assay percentage = (Abs control − Abs treated)/(Abs control) × 100

### 3.12. Anti-Inflammatory Activity

#### 3.12.1. Inhibition of Protein Denaturation

The assay was conducted with minor alterations to the protocol established by [65]. In summary, 0.2 mL of freshly synthesized egg albumin was incorporated into 2.8 mL of PBS, pH 6.5. Subsequently, 2 mL of silver nanoparticles (AgNPs) at varying concentrations (20–100 μg/mL) were introduced. The resultant reaction mixture (5 mL) was subjected to incubation at 37 ± 1 °C for 30 min, followed by heating at 70 ± 1 °C for 5 min. Upon cooling to ambient temperature, the absorbance was measured at 660 nm utilizing a UV–visible spectrophotometer. An analogous configuration was employed for the reference drug (diclofenac sodium). The percentage inhibition of protein denaturation was determined utilizing the subsequent equation:

INC% = (D_t_ − D_0_)/D_0_ × 100

where

D_t_: paw diameter at t time;

D_0_: initial paw diameter.

#### 3.12.2. Membrane Stability Assay

The capacity for membrane stabilization of the synthesized AgNPs was evaluated employing the human red blood cell (HRBC) methodology as outlined by [66], albeit with slight modifications. Fresh human blood was obtained from a healthy donor and mixed with an equivalent volume of Alsever’s solution. The resulting mixture was centrifuged at 3000 rpm for a period of 10 min, after which the packed erythrocytes were meticulously rinsed three times with isotonic saline. Thereafter, a 10% suspension was formulated. For the assay, 1 mL of AgNPs at varying concentrations (20–100 μg/mL), 1 mL of phosphate buffer (pH 7.4), 1 mL of hyposaline (0.25% NaCl), and 0.5 mL of the 10% HRBC suspension were amalgamated. The resulting tubes were incubated at a regulated temperature of 37 ± 1 °C for 30 min, followed by centrifugation at 3000 rpm for an additional 10 min. The absorbance of the resulting supernatant was measured at a wavelength of 560 nm. The percentage of membrane stabilization was calculated using the following formula:

INC % = (D_t_ − D_0_)/D_0_ × 100

where

D_t_: paw diameter at t time;

D_0_: initial paw diameter.

### 3.13. Statistical Analysis

Statistical assessments were performed employing one-way analysis of variance (ANOVA). The statistical computations were executed using SPSS, version 17.0. Three independent replications for each experimental and assay procedure were performed (*n* = 3). The average of the three values was reported in each instance.

## 4. Conclusions

This investigation effectively elucidated CG-AgNPs utilizing *C. gigantea* leaf extract as a reducing and stabilizing agent. The resultant nanoparticles exhibited distinctly characterized physicochemical attributes and substantial bioactivity. Importantly, CG-AgNPs demonstrated pronounced antibacterial efficacy against *H. pylori*, including strains resistant to multiple drugs, underscoring their potential as an innovative antimicrobial agent. Moreover, the nanoparticles manifested exceptional antioxidant activity, proficient inhibition of pro-inflammatory markers, and significant cytotoxicity against gastric carcinoma cells (AGS). The multifaceted therapeutic effects of CG-AgNPs indicate their robust potential as a multifunctional agent for the therapeutic intervention of gastric infections associated with *Helicobacter pylori* and inflammation-induced carcinogenesis. In light of their environmentally friendly synthesis, minimal toxicity, and broad-spectrum efficacy, these nanoparticles present a promising alternative to traditional therapeutic approaches. Subsequent in vivo investigations and mechanistic elucidation at the molecular level will be imperative to translate these findings into clinical applications. Nevertheless, this research establishes a robust foundation for the advancement of plant-derived nanoparticle therapeutics in addressing *H. pylori*-related gastric disorders and beyond.

## Figures and Tables

**Table 1:** The GC/MS analysis of the aqueous leaf extract of *C. gigantea*.

SI NO	RT	Compound Name	Formula	M.WT	Area %
1	6.812	Calein A	C22H28O8	420	4.74
2	7.299	Methyl N-(N-benzyloxycarbonyl-beta-l-aspartyl)-beta-d-glucosaminide	C19H26N2O10	442	2.73
3	7.426	3-(2,5-Bis(benzyloxy)phenyl)-2-formylaminoacrylic acid, benzyl ester	C31H27NO5	493	7.56
4	7.944	[(3aR,4R,5R,6E,9Z,11aR)-4-Hydroxy-6,10-dimethyl-3-methylidene-2,8-dioxo	C22H26O7	402	4.68
5	8.809	Esmolol, 2TMS derivative	C16H26ClNO4	439	5.35
6	8.809	Di-carobenzyloxy-l-4-hydroxylysine	C22H26N2O7	430	2.37
7	8.931	Cholestan-3-amine, N, N,4,4-tetramethyl-, (3. beta.,5.alpha.)-	C31H57N	443	3.64
8	9.117	2-Heptafluorobutyroxytetradecane	C18H29F7O2	410	3.42
9	9.186	2-Phenylbutyramide, N-octadecyl-	C28H49NO	415	4.13
10	9.361	3-(4-Chlorobenzylideneamino)-N-[5-oxo-1-(2,4,6-trichlorophenyl)-2-pyrazolin-3-yl] benzamide 3-([(E)-(4-Chlorophenyl) methylidene] amino)-N-[5-oxo-1-(2,4,6-trichlorophenyl)-4	C23H14Cl4N4O2	518	3.06
11	10.357	4,6-Bis(4-ethoxybenzylthio)-5-nitropyrimidine	C22H23N3O4S2	457	1.80
12	10.750	2-[2-[2-[2-[2-[2-[2-[2-(2-Methoxyethoxy) ethoxy] ethoxy] ethoxy] ethoxy] ethoxy] ethoxy] ethoxy] ethyl acetate $$ Nonaethylene glycol monomethyl ether, acetate	C21H42O11	470	1.25
13	11.074	Heptafluorobutyric acid, n-tetradecyl ester	C18H29F7O2	410	2.28
14	11.200	(1R,3R,3aS,4S,7R,9aR,10aR, Z)-1-(Acetoxymethyl)-7-isopropyl-4,9a-dimethyl	C26H40O6	448	2.02
15	11.585	Melezitose. alpha. -D-Glucopyranoside, O-. alpha. -D-glucopyranosyl-(1. fwdarw.3)-. beta. -D-fructofuranosyl Glucopyranoside, O-. alpha.-D-glucopyranosyl	C18H32O16	504	4.74
16	12.016	Tetracosanoic acid, isopropyl ester	C27H54O2	410	1.37
17	12.080	Octacosane, 2-methyl-	C29H60	408	6.19
18	12.721	[1,2-Bis[5-phenyl-2,4-pentadienoyl] hydrazino] acetic acid ethyl ester	C26H26N2O4	430	1.88
19	12.773	(Z)-Tetradec-11-en-1-yl 2,2,3,3,4,4,4-heptafluorobutanoate	C18H27F7O2	408	3.27
20	12.833	Heptacosane, 1-chloro-1-chloroheptacosane	C27H55Cl	414	1.13
21	15.392	Phthalic acid, isobutyl tridec-2-yn-1-yl ester	C25H36O4	400	2.02
22	15.882	Phthalic acid, butyl tridecyl ester	C25H40O4	404	2.77
23	16.107	8-(1,2-Dihydroxy-3-methyl-3-butenyl)-7-methoxy coumarin,	C15H16O5	276	10.60
24	16.142	Bis[phenylsulfonyl]-4-carboxyl phenyl methane	C20H16O6S2	416	1.33
25	16.328	Bis[phenylsulfonyl]-4-carboxyl phenyl methane	C20H16O6S2	416	7.43
26	16.485	Phthalic acid, butyl tridec-2-yn-1-yl ester	C25H36O4	400	4.44
27	16.652	Phthalic acid, 4,4-dimethylpent-2-yl hexadecyl ester	C31H52O4	488	1.56
28	18.329	alpha-amyrin	C30H50O	426	1.17
29	19.948	Phthalic acid, 6-ethyloct-3-yl 2-ethylhexyl ester	C26H42O4	418	1.08

**Table 2:** FTIR spectral band assignments of green-synthesized CG-AgNPs derived from *Calotropis gigantea* leaf extract, indicating the specific functional groups that play a role in the processes of nanoparticle reduction and stabilization.

Sl. No	Wavelength Number cm^−1^	Stretching Vibrations	Functional Groups
1	3426.01	O–H stretching	Phenolic groups or alcohols
2	2924.07	C–H stretching	Alkanes
3	2852.37	C–H stretching	Alkanes
4	1596.39	CH_2_ and CH_3_ bending	Alkanes
5	1456.65	N–H bending	Amines
6	1216.09	C–N stretching	Amines
7	1025.20	C–O stretching	Carboxylic acids
8	777.47	C–Cl or C–Br stretching	Alkyl halides
9	618.23	C–Cl or C–Br stretching	Alkyl halides

## Data Availability

The original contributions presented in this study are included in the article. Further inquiries can be directed to the corresponding authors.
